# News and Notes

**Published:** 1902-09

**Authors:** 


### NEWS AND NOTES.

(We are indebted to the *Medical Age* for many of these notes.)

---

THERE are about 25,000 hospitals and asylums in the United States.

---

THE cruiser Dixie is to be transformed into a hospital ship at a cost of about $75,000.

---

THE ninth international congress against the abuse of alcohol will be held at Bremen, April 14 to 19, 1903.

---

AN appropriation of $500,000 has been made for the erection in New York of a hospital for contagious diseases.

---

THE Rush Medical College has decided to allow women to matriculate for graduation upon the same footing as men.

---

THE Archduke Carl Theodore, of Bavaria, the distinguished ophthalmic surgeon, recently performed his four-thousandth operation for cataract.

---

ACCORDING to press reports ninety American soldiers have died of cholera in the Philippines since the disease first made its appearance among them.

---

DR. G. H. Simmons, the valued editor of the *Journal of the American Medical Association*, was recently operated on for gall-stones and is now convalescent.

---

WE have received the first number of the *Old Dominion Journal of Medicine and Surgery*. It is published by the Alumni Society of the Medical College of Virginia, and is edited by George Baughman, M.D., and A. B. Greiner, M.D. We wish it success.

---

At a recent meeting of the Mobile County Medical Society, an appropriation of one hundred dollars was made as the society's contribution for the Jerome Cochran monument. This is an act that reflects creditably upon the society and should stimulate the the other societies in the State to imitate it.

---

The Tri-State Medical Society of Alabama, Georgia and Tennessee will convene in Birmingham October 7, 1902. Judging from the preliminary program there will be a large number of interesting papers which will be read and discussed at this meeting. The Society will be in session three days, and the medical profession of these States is especially invited to attend the meeting and take part in its proceedings.

---

During the last thirty years a number of physicians have served in France as a chiefs of the various state departments, public instruction, etc. The President of the Republic has recently appointed Dr. E. Combs to preside over the "Conseil des ministres." He is minister of the interior, and had previously served as minister of public instruction, after leaving his practice at Pons for a place in the senate in 1885. Our French exchanges observe that he was not selected on account of his profession, but that nevertheless it is a satisfaction to know that one of its members is qualified for this important post, and has been thus honored. The French legislature has now fifty-five medical members—nine per cent. of the total number.—*Exchange.*

---

The Tricounty Medical Association of Cobb, Douglas and Paulding counties held its first regular quarterly meeting at Black's hall in Marietta August 13.

The meeting was presided over by Dr. C. T. Nolan, president of the Association.

There was a good attendance by representative physicians from each of the counties. A regular program which had been previously prepared was carried out and several interesting practical papers were read and discussed.

The visiting physicians were entertained at luncheon at the Kennesaw house by the local physicians. The meeting was in every respect a most delightful and instructive one, and its members are very enthusiastic over the future prospects of the Association.

Following is the program carried out:

"The Treatment of Summer Diseases of Childhood," by Dr. J. D. Malone, of Marietta, Ga.

"A Few Remarks on Diagnosis, with Report of Cases," by Dr. J. D. Middlebrooks, of Powder Springs.

"Typhoid Fever and its Treatment Twenty-five Years Ago Compared to Present Methods," by Dr. J. L. Selman, of Douglasville.

"A report of Some Surgical Cases," by Dr. C. T. Nolan, Marietta, Ga.

"The Indiscriminate Use of Coal-tar Antipyretics," by Dr. W. L. Wright, Powder Springs.

The next meeting will be held the second Wednesday in November, at Douglasville, Ga.

---

THE Fourteenth International Congress of Medicine will be held in Madrid, Spain, from April 23d to 30th, 1903, under the patronage of their Majesties, the King of Spain and the Queen-Mother. The President of the Congress is Professor Julian Callejay Sanchez, the General Secretary is Dr. Angel Fernande-Caro, and the General Treasurer is Professor Jose Gomez Ocana. The preliminary statement of regulations and program has just been issued, and it announces that the members of the Congress will be physicians, pharmacists, dentists, veterinary surgeons, and other persons working at branches of medical science, both Spaniards and foreigners, who have entered their names and paid their subscriptions. Other persons who possess scientific or professional titles, and who wish to take part in the work of the Congress may share in it under the above conditions. The subscription is thirty pesetas, and this sum must be paid before the opening of the Congress to the General Secretary, Faculty of Medicine, Madrid. A card of membership will sent to the subscriber. Until March 20th, 1903, subscriptions may be paid to the Secretary of the National Committee of the subscriber, but after that date subscriptions must be paid directly to the General Secretary at Madrid. Members will receive a summary of the proceedings of the Congress, and a full report of the particular section which they join. The official languages of the Congress will be Spanish, French, English, German and Italian. Papers must be sent to the General Secretary before January 1st, 1903, to be certain of a place in the order of business. Papers presented later will be considered after the discussion of those regularly announced. Communications should be accompanied by a short abstract, which will be printed and distributed among the members of the Congress. J. H. Huddleston, Secretary American Committee, 126 West 85th St., New York City.

